# Major Adverse Limb Events and Death After Successful Endovascular Revascularization: BEST-CLI Trial

**DOI:** 10.1016/j.jscai.2025.104192

**Published:** 2026-02-24

**Authors:** Scott Kinlay, Alik Farber, Matthew T. Menard, Michael B. Strong, Michael D. Dake, John Kaufman, Peter A. Schneider, Michael S. Conte, Palma M. Shaw, Vikram S. Kashyap, Kenneth Rosenfield, Gheorghe Doros, Jeffrey J. Siracuse, Richard J. Powell

**Affiliations:** a Interventional Endovascular Therapy, Cardiovascular Division, Department of Medicine, VA Boston Healthcare System, Boston, Massachusetts; b Harvard Medical School, Boston, Massachusetts; c Cardiovascular Division, Department of Medicine, Mass General Brigham, Boston, Massachusetts; d Division of Vascular Surgery and Endovascular Surgery, Department of Surgery, Boston Medical Center, Boston University Chobanian & Avedisian School of Medicine, Boston, Massachusetts; e Vascular and Endovascular Surgery, Heart & Vascular Center, Brigham and Women's Hospital, Boston, Massachusetts; f Department of Medical Imaging, University of Arizona Health Sciences, Tucson, Arizona; g Department of Interventional Radiology, Oregon Health and Science University, Portland, Oregon; h Division of Vascular and Endovascular Surgery, UCSF Medical Center, San Francisco, California; i Vascular Surgery and Endovascular Services, SUNY Upstate Medical University, Syracuse, New York; j Frederik Meijer Heart and Vascular Institute, Corewell Health, Michigan State University College of Human Medicine, Grand Rapids, Michigan; k Vascular Medicine and Intervention, Division of Cardiology, Massachusetts General Hospital, Boston, Massachusetts; l Boston University School of Public Health, Boston, Massachusetts; m Heart and Vascular Center, Dartmouth Hitchcock Medical Center, Lebanon, New Hampshire

**Keywords:** chronic limb-threatening ischemia, critical limb ischemia, death, endovascular, major adverse limb event, risk factors

## Abstract

**Background:**

Chronic limb-threatening ischemia (CLTI) has a high risk of limb amputation without revascularization. In the Best Surgical Therapy in CLTI (BEST-CLI) trial, endovascular revascularization had a higher risk of major adverse limb events (MALE) or death compared with surgical bypass with a good quality vein. However, endovascular revascularization is still required for patients with poor vein options or high surgical risk. We assessed the factors related to MALE or death among patients with a successful endovascular intervention in the BEST-CLI trial.

**Methods:**

All patients with successful endovascular revascularization in the BEST-CLI trial were followed for a mean of 2.7 years. Baseline patient characteristics, lesion characteristics, and endovascular techniques were compared with the subsequent risk of MALE or death. Multivariable models estimated hazard ratios (HRs) and 95% CIs from Cox proportional hazards models.

**Results:**

Of the 923 patients having endovascular revascularization, 773 (84%) had a successful index procedure. In femoral-popliteal interventions, MALE or death was associated with end-stage renal disease (HR, 1.64; 95% CI, 1.17-2.29), wounds at or above the ankle (HR, 2.13; 95% CI, 1.38-3.29), and longer procedure time (HR, 1.15 per 120 minutes; 95% CI, 1.02-1.30). In below-knee popliteal-tibial interventions, MALE or death was associated with diabetes mellitus (HR, 1.69; 95% CI, 1.18-2.43), end-stage renal disease (HR, 1.80; 95% CI, 1.26-2.57), and longer procedure time (HR, 1.28 per 120 minutes; 95% CI, 1.11-1.47). Interventional technique, including drug-coated technologies, did not relate to MALE or death.

**Conclusions:**

Patient factors were strongly related to MALE or death after successful endovascular revascularization for CLTIs. Endovascular techniques, including drug-coated balloons and stents, were not consistently related to MALE or death in this high-risk population of patients with CLTI, justifying their use when needed for complex disease.

## Introduction

Chronic limb-threatening ischemia (CLTI) is associated with a high risk of major amputation and death.^1^^,^^2^ Revascularization improves limb preservation and maintains functional independence in patients.

There are a multitude of endovascular revascularization options for peripheral artery disease (PAD), including plain balloon angioplasty, drug-coated balloons, drug-eluting stents, bare-metal stents, stent grafts, and atherectomy.^3^ These endovascular therapies are used either as definitive treatment or as adjunctive therapies to modify plaque (eg, atherectomy or laser) in arteries above and below the knee. Recently, 2 major clinical trials, the Best Endovascular versus Best Surgical Therapy in Patients with Clinical Limb Ischemia (BEST-CLI) trial^4^ and the Bypass versus Angioplasty for Severe Ischemia of the Leg - 2 (BASIL-2) trial,^5^ compared infrainguinal surgical bypass versus endovascular revascularization in patients with CLTI who were deemed eligible for both approaches. The studies came to divergent conclusions, with endovascular revascularization having worse limb and survival outcomes than surgical bypass with greater saphenous vein in BEST-CLI, similar outcomes to bypass with other conduits in BEST-CLI, and better outcomes to bypass in BASIL-2. Trying to synthesize these inconsistent results has proved difficult,^6^ with the endovascular community raising questions about endovascular treatment in BEST-CLI, including the severity of arterial disease, the use of advanced endovascular techniques, and the use of drug-eluting stents and balloons.

The objective of this post-hoc analysis of the BEST-CLI trial was to describe the patient and limb characteristics and endovascular techniques used in patients with successful index endovascular procedures. We assessed the relationship of these factors to MALE or death over the duration of the study.

## Methods

This was an as-treated analysis of all the subjects in the BEST-CLI trial (ClinicalTrials.gov number NCT02060630) who had a successful endovascular revascularization as their initial mode of revascularization. Procedural success was defined as the ability to cross the lesion with a wire, a residual stenosis <50%, and in-line flow to the foot in at least 1 tibial artery.^4^ Patients who experienced endovascular technical failure were reported previously and excluded from this analysis.^7^ The trial protocol was approved by the ethics committee at each participating site, and study details were reported in the main results of the BEST-CLI trial.^4^

### Patient population

Patients included were 18 years or older with CLTI defined as arterial insufficiency of the lower limb with ischemic foot pain at rest, a nonhealing ulcer, or gangrene and corroborated with hemodynamic criteria. Patients with successful endovascular revascularization included 750 subjects randomized to endovascular therapy in either of the 2 cohorts, and 23 patients who were randomized to infrainguinal bypass, but who received endovascular revascularization instead. Patient characteristics included risk factors for atherosclerosis, comorbidities, medication use, prior interventions, physical examination, and lower extremity noninvasive studies.^4^

### Limb characteristics

Limb characteristics included the presence of ischemic rest pain and tissue loss, wound location, and the wound, ischemia, and foot infection (WIfI) wound classification.^8^ Procedural factors included access sites, procedural time, the endovascular techniques used, and the use of closure devices. Complications included arterial thrombosis, embolization, dissection, or perforation during the procedure.

**Figure 1:**
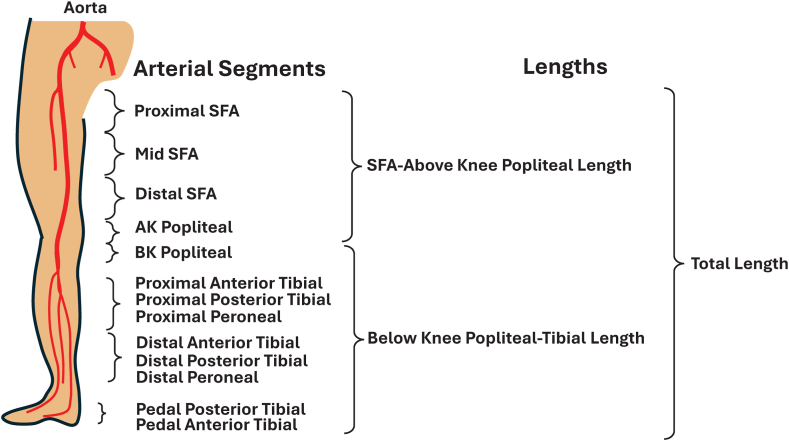
**Arterial segments used to determine lengths in the analysis.** AK, above knee; BK, below knee; SFA, superficial femoral artery.

The lower extremity arterial tree was divided into 3 superficial femoral artery (SFA), 2 popliteal artery, 6 tibial artery, and 2 pedal artery segments (Figure 1). For each segment, the operators visually estimated the most severe stenosis or occlusion and lesion length. Since device use was markedly different for below-knee versus above-knee interventions, we generated a variable termed “SFA-above-knee popliteal artery” and described the most severe stenosis and the sum of lesion lengths treated in these segments. Similarly, a second variable of “below-knee popliteal artery-tibial” described the most severe stenosis and the sum of the lesion lengths treated in these segments. Analyses on device use were stratified by these 2 regions, acknowledging that some patients had interventions in both regions. Total lesion length was the sum of the SFA to above–knee popliteal length and the below-knee-to-popliteal artery-tibial length (Figure 1). In multivariable analyses, lesion lengths for SFA-popliteal lesions and popliteal-tibial lesions were categorized as <100 mm, 100 <200 mm, and >200 mm.

### Endovascular technique

Endovascular techniques used were recorded for every treated infrainguinal arterial segment. Plain angioplasty alone was defined as the use of balloon dilation in any given segment without any additional techniques. Other techniques per segment included any use of plain or cutting balloon angioplasty, bare-metal stent, drug-coated balloon, drug-eluting stent, stent graft, laser atherectomy, other atherectomy, mechanical thrombectomy, or any combination of these techniques. For multivariable analyses, we compared the use versus no use of these devices. The use of luminal reentry devices and embolic protection devices was also recorded.

### Outcomes

The primary outcome for this analysis was MALE or death, with MALE defined as above-ankle amputation of the index limb, or a major index-limb reintervention (new bypass, interposition graft revision, thrombectomy, or thrombolysis).^4^ Major index-limb reinterventions were adjudicated by an independent multidisciplinary clinical-events committee as part of the parent BEST-CLI trial. We also assessed risk factors for all-cause death over follow-up. Patients were followed up to 7 years for a median of 2.7 years (IQR, 1.6-4.0).

### Statistical analysis

Patient, limb, and endovascular techniques were described using means and standard deviations or percent, as appropriate. Differences between patients who did versus who did not have a subsequent MALE or death were assessed using χ^2^ tests for categorical data and summarized as numbers and percentages. Continuous data are reported as means with standard deviations or as medians with interquartile ranges and compared using *t* tests or Kruskal-Wallis tests. Cox proportional hazards models were used to determine univariable and multivariable hazard ratios (HRs) and 95% CI for the outcomes according to patient, cohort 1 or 2, and endovascular factors stratified by patients having interventions above or below the knee. Variables were entered into the multivariable models based on clinical significance or if they had a *P* value < .2 in the descriptive tables. These analyses were exploratory and were not adjusted for multiple tests of significance. A *P* value of <.05 was used for statistical significance. All statistical analyses were performed with SAS version 9.4 (SAS Institute).

## Results

**Figure 2:**
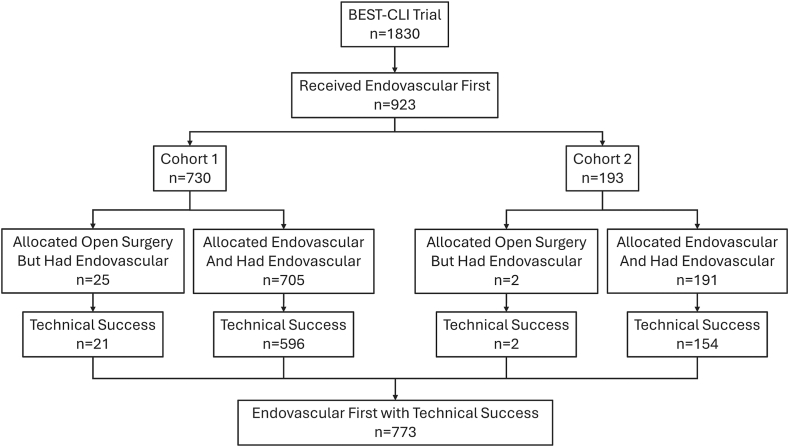
**Flow diagram for defining the cohort of subjects receiving a successful endovascular revascularization as the first procedure**.

Of the 1830 patients enrolled in the BEST-CLI trial, 923 (50%) had endovascular revascularization as their first revascularization treatment, including 27 subjects who were initially randomized to the surgical bypass arm. Of these, 773 (84%) subjects had a successful endovascular revascularization at the index procedure (Figure 2).

### Patient and limb characteristics

Table 1 compares the baseline characteristics of patients who did and did not experience a MALE or death event following successful endovascular therapy. Subjects who experienced a MALE or death event in follow-up were more likely to have diabetes mellitus, heart failure, or end-stage renal disease (ESRD). Table 2 similarly compares the limb and lesion characteristics among patients who did and did not experience a MALE event after endovascular therapy. Of all patients, 95% had WIfI wound stage of 1 or higher, 82% had treatment in the SFA-above-knee popliteal artery territory, and 62% had treatment in the below-knee popliteal artery-tibial territory, and 45% had treatment in both territories. MALE or death was not related to lesion length, total occlusions, total contrast volume, or the use of arterial closure devices (Table 2). However, MALE or death was associated with longer procedure times (*P* = .0004). Complications were rare, but only arterial thrombus was related to a MALE or death (*P* = .012).

**Table 1:** Baseline patient characteristics by subsequent major adverse limb event or death.

Characteristics	MALE or death n = 381	No MALE or death n = 392	*P* value
Age, y	66.9 ± 10.2	66.9 ± 9.6	.73
Female sex	107 (28)	124 (32)	.28
Race			.16
White	266 (70)	287 (74)	
Black	70 (19)	74 (19)	
Other	37 (10)	21 (5)	
Mixed	6 (2)	6 (2)	
Ethnicity			.50
Hispanic	61 (16)	56 (14)	
Non-Hispanic	320 (84)	336 (86)	
BMI, kg/m^2^	28.1 ± 6.0	28.1 ± 6.0	.95
Hypertension	334 (88)	336 (86)	.43
Hyperlipidemia	276 (72)	280 (71)	.75
Diabetes	282 (74)	253 (65)	.004
Current smoking	139 (37)	137 (35)	.66
Prior infrainguinal revascularization of the index limb	23 (6)	23 (6)	.92
Heart failure	33 (9)	14 (4)	.003
Prior stroke	57 (15)	48 (12)	.27
COPD	67 (18)	52 (13)	.10
End-stage renal disease	63 (17)	23 (7)	<.0001
Medications			
Statin	270 (71)	278 (71)	.99
Aspirin	252 (66)	274 (70)	.26
Clopidogrel	99 (26)	99 (25)	.82
Prasugrel	2 (1)	2 (1)	.98
Ticagrelor	8 (2)	2 (1)	.05
Direct oral anticoagulant	18 (5)	15 (4)	.54
Warfarin	27 (7)	26 (7)	.80

Values are mean ± SD or n (%).

BMI, body mass index; COPD, chronic obstructive pulmonary disease; MALE, major adverse limb events.

**Table 2:** Baseline limb and lesion characteristics in patients by subsequent major adverse limb event or death.

Characteristics	MALE or death n = 381	No MALE or death n = 392	*P* value
Ankle-brachial index	0.60 ± 0.31	0.60 ± 0.32	.46
Ischemic rest pain	261 (69)	244 (62)	.07
Tissue loss	152 (78)	431 (75)	.29
Worst wound location			.19
No wounds	69 (18)	91 (24)	
Any toe wound	187 (50)	194 (50)	
Forefoot or hindfoot wound	81 (22)	77 (20)	
Ankle or above-ankle wound	38 (10)	23 (6)	
WIfI wound stage			.07
0 (no ulcer)	70 (19)	92 (24)	
1 (small shallow ulcer)	158 (43)	169 (44)	
2 (deep ulcer with exposed bone)	112 (30)	105 (28)	
3 (extensive ulcer forefoot/heel)	29 (8)	16 (4)	
Lesion characteristics			
SFA or above-knee popliteal artery	320 (84)	316 (81)	.22
Below-knee popliteal or tibial artery	249 (65)	231 (59)	.07
Total occlusion in SFA to above-knee popliteal artery segment	222 (69)	226 (72)	.40
Total occlusion in below-knee popliteal to tibial artery segment	133 (53)	137 (60)	.56
Total lesion length, mm	245 ± 186	227 ± 171	.21
SFA to above–knee popliteal artery length, mm	188 ± 139	171 ± 132	.12
Below–knee popliteal to tibial artery length, mm	132 ± 137	151 ± 152	.29
Procedural characteristics			
Primary Antegrade access	150 (39)	133 (34)	.12
Total procedure time, minutes	171 ± 101	148 ± 89	.0004
Contrast volume, mL	119 ± 85	115 ± 79	.82
Access closure device			.43
None	140 (37)	116 (30)	
Perclose	60 (16)	60 (15)	
Starclose	26 (7)	29 (7)	
Angioseal	74 (20)	83 (21)	
Mynx	60 (16)	76 (19)	
Other	15 (4)	21 (5)	
Complications			
Arterial thrombus	16 (4)	5 (1)	.012
Arterial embolization	12 (3)	14 (4)	.75
Flow-limiting dissection	39 (10)	36 (9)	.62
Perforation or Rupture	7 (2)	16 (4)	.07

Values are mean ± SD or n (%).

WIfI, wound, ischemia, and foot infection.

### Endovascular techniques

Table 3 shows the use of various endovascular techniques in combinations of different arteries by patient treated. Treatments in multiple arteries were common, with treatment of all 3 arteries (SFA, popliteal, and tibial) in 182 (24%) patients, and 2 arteries in 329 (43%) patients. Plain balloon angioplasty alone was used in only 123 (16%) cases and most commonly in the infrapopliteal arteries. Drug-coated balloons were used in 245 (32%), self-expanding drug-eluting stents were used in 132 (17%), and balloon-expandable drug-eluting stents were used in 89 (12%) patients.

**Table 3:** Endovascular procedure characteristics by artery group.

	Artery group							Total
	SFA alone	Popliteal alone	Tibial/peroneal alone	SFA and popliteal	SFA and tibial/peroneal	Popliteal and tibial/peroneal	SFA and popliteal and tibial/peroneal	
N	139	18	104	184	64	81	182	772^a^
Angioplasty alone^b^	10 (7)	1 (6)	63 (61)	8 (4)	6 (9)	16 (20)	19 (10)	123 (16)
Angioplasty—standard balloon^b^	84 (61)	10 (56)	97 (93)	121 (66)	60 (94)	72 (89)	168 (92)	612 (79)
Angioplasty - cutting balloon	8 (6)	0 (0)	5 (5)	9 (5)	3 (5)	3 (4)	13 (7)	41 (5)
Angioplasty drug-coated balloon	42 (30)	6 (33)	5 (5)	66 (36)	17 (27)	39 (48)	70 (39)	245 (32)
Stent bare–metal self- expanding	66 (48)	6 (33)	0 (0)	93 (51)	28 (44)	17 (21)	82 (45)	292 (38)
Stent bare–metal balloon expandible	10 (7)	2 (11)	3 (3)	18 (10)	3 (5)	0 (0)	18 (10)	54 (7)
Stent drug–eluting self-expanding	34 (25)	2 (11)	6 (6)	36 (20)	13 (20)	6 (7)	35 (19)	132 (17)
Stent drug–eluting balloon expandible	14 (10)	0 (0)	17 (16)	6 (3)	10 (16)	10 (12)	32 (18)	89 (12)
Any drug eluting/coated device	80 (58)	8 (44)	28 (27)	98 (53)	31 (48)	48 (59)	112 (62)	405 (53)
Stent graft self-expanding	16 (12)	2 (11)	1 (1)	32 (17)	5 (8)	1 (1)	7 (4)	64 (8)
Stent graft balloon expandible	3 (2)	2 (11)	0 (0)	8 (4)	1 (2)	0 (0)	1 (1)	15 (2)
Laser	2 (1)	1 (6)	0 (0)	4 (2)	1 (2)	3 (4)	4 (2)	15 (2)
Luminal reentry device	5 (4)	0 (0)	0 (0)	3 (2)	0 (0)	0 (0)	3 (2)	11 (1)
Mechanical Thrombectomy	0 (0)	1 (6)	0 (0)	3 (2)	4 (6)	1 (1)	7 (4)	16 (2)

Angioplasty alone includes standard balloon or cutting or scoring balloon with no other interventions. Several of the other interventions could have occurred in any artery, thus the total number of treatments exceeds the numbers of subjects in each arterial group.

Values are n (%).

a One subject had plain balloon angioplasty to the common femoral artery.

b Angioplasty alone = no other device used. Angioplasty—standard balloon = balloon angioplasty with another treatment.

Table 4 shows the endovascular techniques used at the index revascularization divided into above- and below-knee interventions according to subsequent MALE or death. The most common techniques in the SFA-above-knee popliteal territory, ranked from the highest to the lowest, were bare-metal stent (43%), drug-coated balloons (34%), plain balloon angioplasty (24%), drug-eluting stents (18%), atherectomy (12%), and stent grafts (10%). The most common techniques in the below-knee popliteal-tibial territory were plain balloon angioplasty (74%), drug-coated balloons (18%), atherectomy (12%), bare-metal stents (8%), and drug-eluting stents (5%). Combinations of atherectomy and drug-coated balloons were used in 3% to 8% of lesions, and combinations of drug-coated balloons with bare-metal stents in 0% to 10% of lesions. At least 1 drug-eluting/coated technology was used in 330 (52%) interventions in the SFA-above-knee popliteal interventions and 157 (33%) below-knee popliteal to tibial interventions.

**Table 4:** Univariable associations for MALE or death according to endovascular techniques used to treat lesions in the superficial femoral to above–knee popliteal artery and the below–knee popliteal to tibial artery segments.

	MALE or death	No MALE or death	*P* value
SFA to above–knee popliteal artery	n = 320	n = 316	
Any plain angioplasty	84 (26)	69 (22)	.19
Any atherectomy	40 (13)	32 (10)	.35
Any bare-metal stent	136 (43)	132 (42)	.85
Any drug-coated balloon	108 (34)	104 (33)	.82
Any drug-eluting stent	58 (18)	54 (17)	.73
Any drug-coated/eluting device	168 (53)	162 (51)	.76
Any stent graft	34 (11)	25 (8)	.24
Any laser	12 (4)	2 (1)	.007
Any mechanical thrombectomy device	8 (3)	3 (1)	.22
Any luminal reentry device	1 (0.3)	8 (3)	.02
Combination treatment			
Atherectomy with drug-coated balloon	24 (8)	21 (7)	.67
Drug-coated balloon with bare-metal stent	34 (11)	21 (7)	.07
Below-knee popliteal artery to tibial artery	n = 249	n = 231	
Any plain angioplasty	182 (73)	176 (76)	.44
Any atherectomy	31 (12)	26 (11)	.69
Any bare-metal stent	21 (8)	15 (7)	.42
Any drug-coated balloon	42 (17)	44 (19)	.53
Any drug-eluting stent	16 (6)	10 (4)	.31
Any drug-coated/eluting device	84 (34)	73 (32)	.62
Any stent graft	1 (0.4)	4 (2)	.20
Any mechanical thrombectomy device	8 (3)	2 (1)	.11
Any luminal reentry device	1 (0.4)	1 (0.4)	>.99
Combination treatment			
Atherectomy with drug-coated balloon	8 (3)	9 (4)	.81
Drug-coated balloon with bare-metal stent	5 (2)	0 (0)	.06

Multiple combinations used in some patients.

Values are n (%).

In the SFA-above-knee popliteal artery territory, MALE or death events were higher in patients having laser atherectomy compared with no laser atherectomy (4% vs 1%, *P* = .007) and lower with the use of luminal reentry devices (0.3% vs 3%, *P* = .02), but these techniques were used rarely. In the below-knee popliteal artery-tibial territory, MALE or death was not associated with any endovascular technique.

Subjects in cohort 1 had a similar risk of MALE death compared with those in cohort 2 (HR, 0.98, 95% CI, 0.68, 1.41). Therefore, this variable was not included in the multivariable models.

### Multivariable models

Table 5 shows HR from the multivariable analyses for the primary outcome of MALE or death related to patient, limb, and procedural characteristics among patients who had 1 or more lesions treated in the SFA-above-knee popliteal artery segments or the below-knee popliteal-tibial artery segments. For patients with above-knee interventions, the risk of MALE or death was higher with ESRD (HR, 1.64, 95% CI, 1.17, 2.29), a wound at or above the ankle (HR, 2.13, 95% CI, 1.38, 3.29), longer procedural times (HR, 1.15 per 120 minutes, 95% CI, 1.02, 1.03), angioplasty alone (HR, 1.38, 95% CI, 1.04, 1.83), and atherectomy or laser (HR, 1.59, 95% CI, 1.05, 2.41). For patients with below-knee interventions, the risk of MALE or death was higher with diabetes (HR, 1.69, 95% CI, 1.18, 2.43), ESRD (HR, 1.80, 95% CI, 1.26, 2.57), a wound above the ankle (HR, 1.90, 95% CI, 1.10, 3.27), and longer procedure times (HR, 1.28 per 120 minutes, 95% CI, 1.11, 1.47).

**Table 5:** Multivariable risk of major adverse limb events (MALE) or death according to patient factors in 636 patients having successful superficial femoral-popliteal artery endovascular interventions and 480 patients having successful popliteal-tibial artery endovascular interventions.

Variable	MALE or death	
	Superficial femoral-popliteal artery (n = 636)	Popliteal-tibial artery (n = 480)
No. of events	320	249
Patient factors		
Age, per y	0.99 (0.98-1.01)	1.00 (0.99-1.02)
Race		
White	Reference	Reference
Black	1.08 (0.79-1.47)	0.96 (0.69-1.34)
Mixed	0.38 (0.09-1.56)	0.71 (0.25-2.01)
Other	1.26 (0.84-1.88)	1.09 (0.71-1.66)
Diabetes	1.26 (0.97-1.65)	1.69 (1.18-2.43)^a^
End-stage renal disease	1.64 (1.17-2.29)^a^	1.80 (1.26-2.57)^a^
Current smoker	1.09 (0.85-1.39)	1.26 (0.91-1.73)
Worst wound location		
Ankle or above vs none	2.13 (1.38-3.29)^a^	1.90 (1.10-3.27)^b^
Any toe vs none	1.03 (0.76-1.40)	0.80 (0.56-1.16)
Fore/hind foot vs none	1.20 (0.83-1.72)	0.78 (0.52-1.19)
Procedural factors		
Procedure time at 120 minutes	1.15 (1.02-1.03)^b^	1.28 (1.11-1.47)^a^
SFA-popliteal length		
100-200 mm vs <100 mm	0.92 (0.68-1.26)	1.09 (0.79-1.50)
>200 mm vs <100 mm	1.04 (0.79-1.37)	0.83 (0.59-1.18)
Angioplasty alone	1.38 (1.04-1.83)^b^	0.87 (0.60-1.26)
Bare-metal stent	1.19 (0.88-1.60)	1.07 (0.62-1.87)
DCB	1.02 (0.73-1.43)	0.74 (0.49-1.14)
Drug-eluting stent	1.15 (0.83-1.59)	1.15 (0.66-2.03)
Stent graft	1.47 (1.00-2.18)	0.25 (0.03-1.87)
Atherectomy or laser	1.59 (1.05-2.41)^b^	1.16 (0.75-1.79)
Atherectomy and DCB	0.74 (0.40-1.36)	1.11 (0.43-2.85)
BMS and DCB	1.06 (0.65-1.75)	2.51 (0.82-7.67)
Mechanical thrombectomy	1.91 (0.92-3.94)	1.65 (0.75-3.67)

Values are hazard ratio (95% CI).

DCB, drug-coated balloon; SFA, superficial femoral artery.

a *P* < .01.

b *P* < .05.

Table 6 shows the HR from the multivariable analyses for all-cause death. For patients with above-knee interventions, the risk of death was higher for greater age (HR =1 .03 per year, 95% CI, 1.02, 1.05), ESRD (HR, 2.36, 95% CI, 1.61, 3.47), a wound in the fore/hind foot (HR, 1.70, 95% CI, 1.09, 2.67), and longer procedural time (HR, 1.19 per 120 minutes, 95% CI, 1.02, 1.40). For patients with below-knee interventions, the risk of death was higher for greater age (HR, 1.04 per year, 95% CI, 1.02, 1.05), ESRD (HR, 2.60, 95% CI, 1.74, 3.89), and longer procedural time (HR, 1.20 per 120 minutes, 95% CI, 1.01, 1.41). The use of bare-metal stents with drug-coated balloon use was also associated with a higher risk below the knee (HR, 4.77, 95% CI, 1.18, 19.38), but the wide CI indicate the small number of patients with this intervention.

**Table 6:** Multivariable risk of all-cause death according to patient factors in 636 patients having successful superficial femoral-popliteal artery endovascular interventions and 480 patients having successful popliteal-tibial artery endovascular interventions.

Variable	All-cause death	
	Superficial femoral-popliteal artery n = 636	Popliteal-tibial artery n = 480
The No. of events	209	175
Patient factors		
Age, per y	1.03 (1.02-1.05)∗	1.04 (1.02, 1.05)∗
Race		
White	Reference	Reference
Black	1.26 (0.85-1.86)	0.91 (0.61-1.37)
Mixed	0.81 (0.19-3.37)	1.05 (0.36-3.10)
Other	1.11 (0.67-1.85)	1.24 (0.75-2.03)
Diabetes	1.37 (0.97-1.94)	1.40 (0.89-2.21)
End-stage renal disease	2.36 (1.61-3.47)^a^	2.60 (1.74-3.89)^a^
Current smoker	1.09 (0.79-1.51)	1.07 (0.72-1.60)
Worst wound location		
Ankle or above vs none	1.66 (0.92-2.97)	1.92 (0.93-3.99)
Any toe vs none	1.02 (0.68-1.53)	1.14 (0.69-1.87)
Fore/hind foot vs none	1.70 (1.09-2.67)^b^	1.54 (0.90-2.63)
Procedural factors		
Procedure time at 120 minutes	1.19 (1.02-1.40)^b^	1.20 (1.01-1.41)^b^
SFA-popliteal length		
100-200 mm vs <100 mm	0.86 (0.58-1.26)	0.97 (0.65-1.45)
>200 mm vs <100 mm	0.91 (0.64-1.29)	0.57 (0.37-0.89)
Angioplasty alone	1.41 (0.99-2.00)	1.24 (0.79-2.29)
Bare-metal stent	1.05 (0.72-1.53)	1.42 (0.76-2.68)
DCB	1.13 (0.74-1.73)	1.04 (0.63-1.72)
Drug-eluting stent	0.89 (0.58-1.37)	1.25 (0.60-2.58)
Stent graft	1.25 (0.76-2.06)	0.62 (0.08-4.72)
Atherectomy or laser	1.06 (0.60-1.87)	1.34 (0.79-2.29)
Atherectomy and DCB	0.79 (0.35-1.80)	0.35 (0.07-1.70)
BMS and DCB	1.27 (0.68-2.37)	4.77 (1.18-19.38)^b^
Mechanical thrombectomy	1.04 (0.37-2.93)	0.80 (0.23-2.79)

Values are hazard ratio (95% CI).

DCB, drug-coated balloon; SFA, superficial femoral artery.

a *P* < .01.

b *P* < .05.

Overall, the patient characteristics were more strongly and consistently related to adverse outcomes than the endovascular techniques. Multivariable models excluding procedure time yielded similar results.

## Discussion

This analysis of patients who had successful endovascular index revascularization in the BEST-CLI trial describes the wide variety of endovascular techniques that were used. Patients with successful endovascular procedures in the trial had a wide range of lesion lengths, and many procedures required advanced endovascular techniques, including drug-coated balloons and stents. Although some experienced endovascular specialists may have used more advanced techniques, including drug-coated technologies or completed endovascular procedures in a shorter time (which was consistently associated with outcomes), we found no consistent effect of the devices on MALE or death in the BEST-CLI study. Overall, patient factors rather than procedural factors were more strongly associated with adverse outcomes.

### Patient and wound factors

Patient factors associated with MALE or death included diabetes mellitus, ESRD, and a wound at or above the ankle. These factors have also been identified in previous studies.^2^^,^^9^^,^^10^ However, the WIfI wound stage was not related to MALE or death, in contrast to other studies of endovascular revascularization for PAD, where it was associated with poorer healing and mortality.^11^, ^12^, ^13^, ^14^ Other studies show that wound severity is the most important factor in the WIfI score for determining the risk of adverse limb outcomes and mortality.^15^^,^^16^ Our findings may reflect differences in the patient population or wound care between BEST-CLI and other observational studies. For example, the BEST-CLI study required subjects to have a life expectancy of more than 2 years and have an acceptable risk for open surgical revascularization. These criteria would have excluded many patients with a high risk of death because of comorbidities, frailty, poor nutrition, or lack of distal arterial targets who are more commonly in the highest WIfI grades.^17^, ^18^, ^19^

### Lesion and procedural factors

Unlike other studies,^2^^,^^20^^,^^21^ we found that more complex vascular disease, manifested as longer lesion lengths and chronic total occlusions of the SFA-above-knee popliteal segment, was not associated with MALE or death in univariable or multivariable analyses. This may relate to the method of assessing lesion length, which was based on visual estimates by the operators. That stated, our results are consistent with a prior study where lesion length was related to repeat revascularization, but not MALE.^22^

MALE or death after SFA-above-knee popliteal artery interventions occurred more often in patients having angioplasty alone, atherectomy or laser, and stent graft placement. Atherectomy is associated with a higher risk of distal embolization in large registries.^23^^,^^24^ However, the use of these devices is also more common in complex and calcified diseases.^22^^,^^25^ Stent grafts used above the knee were also associated with a higher risk of major reintervention and an increased risk of MALE. Meta-analyses suggest that covered stents offer no benefit in preventing MALE^26^ and may be associated with worse outcomes than drug-eluting stents.^27^ However, like atherectomy, they are used for arterial perforation, and their use may reflect more complex disease or procedures. Despite these findings, MALE was not higher in patients having these endovascular procedures in the below-knee-tibial region, and none were related to all-cause death in either region. The lack of consistent relationships to these end points and the potential for reverse causation (complex lesions require advanced techniques) support the use of adjunctive therapies if they are required for optimal plaque modification (Graphic Abstract).

Drug-coated balloons or drug-eluting stents were used in 52% of SFA-above-knee popliteal interventions and 33% of below-knee popliteal artery to tibial interventions. During the BEST-CLI trial, there were concerns about the safety of drug-coated technologies, and there were no approved drug-coated balloons for use below the knee in the United States. These likely limited their overall use in the trial, although their use was comparable with data from national registries reporting over a similar time as the trial.^28^^,^^29^ The efficacy of drug-coated technologies is less well known for CLTI, but as there was no consistent difference in the risk of MALE or all-cause death with drug-coated/eluting devices above or below the knee, this adds to more recent trial data and registries supporting their safety.^30^, ^31^, ^32^, ^33^, ^34^, ^35^, ^36^, ^37^, ^38^ Although short-term studies suggest improved patency with drug-eluting technologies used in below-the-knee endovascular revascularization,^39^, ^40^, ^41^ their efficacy with longer follow-up requires further elaboration.^42^ Our analysis did provide some evidence to support the use of drug-coated balloons after atherectomy, which had a significantly lower risk of MALE compared with atherectomy alone in the SFA-popliteal artery distribution. Although this combination is intuitively attractive, other studies have failed to show long-term benefit compared with drug-coated balloons alone.^43^^,^^44^

### Markers of extent and complexity of disease

A unifying hypothesis to explain the patient and procedural factors associated with adverse limb events could be their relationship to the extent and complexity of macrovascular and microvascular PAD. Diabetes mellitus and ESRD are well known to impair the microcirculation and cause wounds disproportionally to other atherosclerosis risk factors.^45^, ^46^, ^47^ Other studies indicate that wounds and other factors related to microvascular disease may be more important determinants of major adverse limb outcomes in CLTI.^22^ Long procedural times and atherectomy could be markers for more diffuse or calcified atherosclerosis, typical of more advanced stages of disease, which in itself is related to a higher risk of adverse limb outcomes.^23^^,^^24^^,^^48^ Thus, differences in outcomes between endovascular techniques likely reflect the use of some technologies (eg, atherectomy) in more complex disease (Central Illustration).

**Central Illustration:**
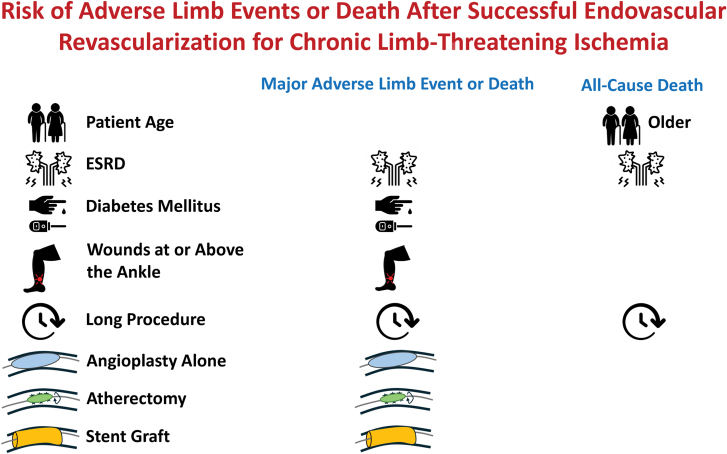
**Factors related to adverse outcomes after successful endovascular revascularization for chroniclimb-threateningischemia (CLTI) in theBEST-CLIstudy.** ESRD, end-stage renal disease.

### Limitations

This analysis of the BEST-CLI study was an observational design, and the results may include unknown confounders. The pragmatic design of the trial, incorporating a wide range of permutations of endovascular devices, renders it challenging to make direct comparisons of individual device efficacy or assess the numerous permutations of different combinations of treatment. However, we were able to describe and assess the risk of some of the more common combinations of devices. The limitations of visual estimates of lesion length and complexity will be addressed in a recently funded assessment of baseline angiograms from the BEST-CLI study. Some associations may be affected by confounding, for example, devices such as atherectomy, stent grafts, and mechanical thrombectomy are likely indicators of more complex and/or calcified disease. As such, their relationships to adverse limb outcomes may reflect the extent and severity of the underlying disease rather than the direct effects of these devices. Similarly, longer procedure times could also reflect lesion complexity or operator experience in endovascular procedures.

## Conclusions

A wide variety of endovascular techniques and lesion severity were used in cases of successful endovascular revascularization in the BEST-CLI study. The relationships of patient and endovascular techniques to adverse outcomes likely reflect the extent and severity of PAD and identify patients who may benefit from closer surveillance after endovascular revascularization. There were no consistent relationships between the endovascular techniques, including drug-coated balloons and drug-eluting stents, and major adverse limb outcomes or death in this study of high-risk patients with CLTI.
